# Rare presentation of an atrial myxoma in an adolescent patient: a case report and literature review

**DOI:** 10.1186/s12887-018-1313-6

**Published:** 2018-11-28

**Authors:** Eduardo Macias, Elizabeth Nieman, Kentaro Yomogida, Orlando Petrucci, Cylen Javidan, Kevin Baszis, Shafkat Anwar

**Affiliations:** 1 Division of Cardiology, Department of Pediatrics, Washington University School of Medicine in St. Louis, One Children’s Place, Campus Box 8116-NWT, St. Louis, MO 63110 USA; 2 Division of Dermatology, Department of Medicine, Washington University School of Medicine, St. Louis, MO USA; 3 Division of Rheumatology, Department of Pediatrics, Washington University School of Medicine, St. Louis, MO USA; 4 Division of Cardiothoracic Surgery, Department of Surgery, Washington University School of Medicine, St. Louis, MO USA; 5 Mallinckrodt Institute of Radiology, Washington University School of Medicine, St. Louis, MO USA

**Keywords:** Myxoma, Purpuric rash, Systemic symptoms, Neurological sequelae, Paraneoplastic vasculitis, Embolic phenomena

## Abstract

**Background:**

Cardiac tumors are uncommon in the pediatric population. When present, cardiac manifestations stem from the tumor causing inflow or outflow obstruction. While common in adults, cardiac myxomas presenting with generalized systemic illness or peripheral emboli especially with no cardiac or neurological symptoms are rare in children.

**Case presentation:**

We report a case of a previously healthy adolescent girl who presented with a 6-month history of constitutional symptoms and a purpuric rash with no cardiac or neurologic symptoms, found to have a cardiac myxoma.

**Conclusions:**

A vasculopathic rash in the setting of atrial myxomas has been shown be a precursor to significant morbidity and mortality. Due to the rarity of this entity, the time elapsed from onset of non-cardiac symptoms until diagnosis of a myxoma is usually prolonged with interval development of irreversible neurological sequelae and death reported in the literature. Therefore, we highlight the importance of including cardiac myxomas and paraneoplastic vasculitis early in the differential diagnosis for patients presenting with a purpuric rash and systemic symptoms.

## Background

Cardiac tumors are rare in the pediatric population, found in less than 0.5% of children evaluated for cardiac disease [1]. Atrial myxomas are the most common cardiac tumor in adults, most commonly presenting with cardiac symptoms. Embolic phenomena, and constitutional or systemic signs [2, 3] follow cardiac symptoms in frequency. In pediatrics, myxomas comprise less than 15% of cardiac tumors [1, 4–6] and presentation with non-cardiac, constitutional symptoms is rare. Thus, we report a case of an adolescent girl who presented with a rash, fever, purpura, and elevated inflammatory markers found to have to a cardiac myxoma. Our case establishes a unique precedent of a diagnosis of cardiac myxoma manifesting with systemic symptoms and a vasculopathic rash, but no neurological embolic phenomena.

## Case presentation

A 13-year-old previously healthy girl presented to the emergency department (ED) for evaluation of fever, bilateral foot pain, and rash. Her symptoms began 6 months prior to presentation, occurring 1–2 times per month, lasting for 2–3 days, and improving with ibuprofen. A few weeks prior to ED presentation, she noted onset of fatigue, pain in her hip and calf, which she attributed to her competitive soccer playing, and acute abdominal pain with diarrhea and emesis.

Patient denied fever, headaches, visual changes, oral ulcers, muscle pain, or changes in bowel and bladder functions. She had no other medication use or recent travel. Her family history was negative for autoimmune diseases.

**Fig. 1:**
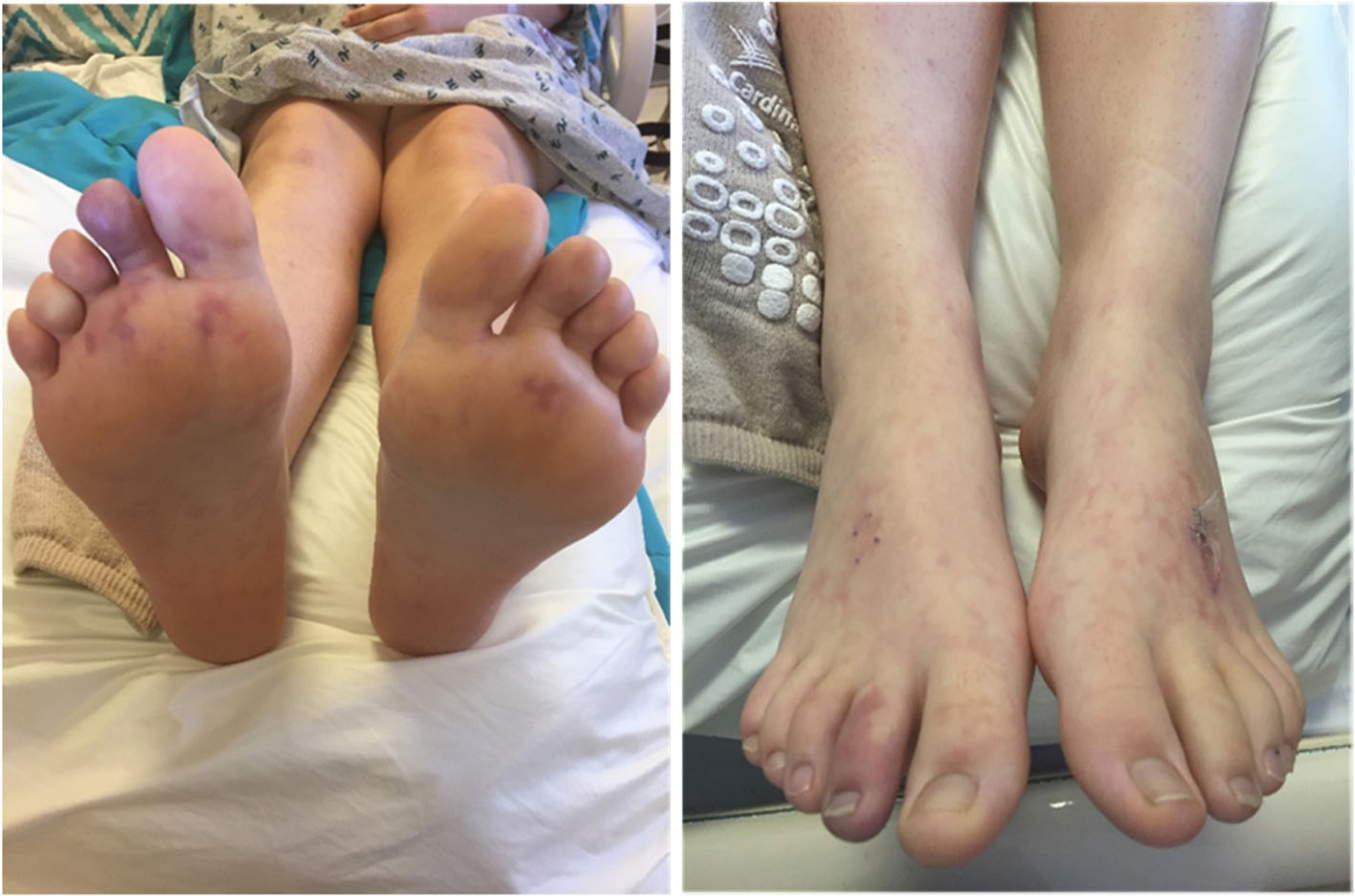
Skin findings at presentation, including retiform patches, purpura and duskiness of right 2nd toe. Left foot dorsum with biopsy sutures in place

Her vital signs upon presentation showed a temperature of 39.2 degrees Celsius, heart rate of 90 beats per minute, respiratory rate of 20 breaths per minute, blood pressure of 114/62 mmHg, and 97% oxygen saturation on room air. She had a blanchable, retiform, violaceous patches with few areas of true purpura on her bilateral lower extremities and duskiness of her right second toe (Fig. 1). The remainder of her physical exam was unremarkable: a regular heart rate and rhythm with normal S1, S2 and no murmurs, rubs, or gallops; 2+ symmetric peripheral pulses; and joints with full range of motion without effusion or warmth. Her neurological exam was normal with no focal deficits. Her inflammatory markers were elevated (Table 1) and she was admitted for further evaluation.

**Table 1:** Inflammatory markers at initial presentation

Laboratory	Value	Reference Range
Creatinine Kinase	1076	< 300 U/L
LDH	1216	100-250 U/L
CRP	48.8	< 10.0 mg/L
ESR	16	< 20.0 mm/hr
AST/ALT	164/89	10-50 U/L; 10-40 U/L
GGT	14	5–35 U/L
IL-6	< 0.5 ng/Ml^a^	

*Abbreviations*: *LDH* lactate dehydrogenase, *CRP* C-reactive protein, *ESR* erythrocyte sedimentation rate, *AST* aspartate transaminase, *ALT* alanine transaminase, *GGT* gamma-glutamyl transferase, *Il* Interleukin

^a^Level drawn 6 days after initial presentation and 4 days after a IV dose of methylprednisolone

Initial diagnostic considerations were vasculitis, including cutaneous polyarteritis nodosa, leukocytoclastic vasculitis (associated with lupus erythematosus, infection or idiopathic), versus a vasculopathy due to antiphospholipid antibody syndrome, cryoglobulinemia, coagulopathy or septic emboli (Table 2).

**Table 2:** Differential diagnosis

Rheumatologic/hematologic	Neoplastic	Infectious	Cardiac
Polyarteritis nodosa	Paraneoplastic vasculitis	Septic emboli	Cardiac myxoma
Leukocytoclastic vasculitis, inflammatory		Leukocytoclastic vasculitis	
Coagulopathy (i.e., antiphospholipid syndrome, cryoglobulinemia, other inherited disorder)			
Henoch-Schonlein purpura			

Skin biopsy of a non-palpable purpuric area on the dorsum of her left foot demonstrated subtle ischemia, vascular congestion, purpura, and focal eccrine gland necrosis without evidence of vasculitis, which was concerning for a vasculopathic process. Direct immunofluorescence of the skin biopsy was negative. Extensive systemic work-up was unremarkable and included: hepatitis panel, cryoglobulins, lupus anticoagulant panel, cardiolipins, beta-2-microglobulin, beta-2-glycopreotin, complements 3 and 4, antistreptolysin O (ASO) titers, anti-DNAse B, and aldolase. Abdominal and renal Doppler ultrasounds were also unremarkable.

During her hospital course, she developed recurrent fevers that were treated with acetaminophen at typical doses. In addition, she received a 1-time dose IV methylprednisolone 1 mg/kg after her battery of laboratory tests and skin biopsy.

**Fig. 2:**
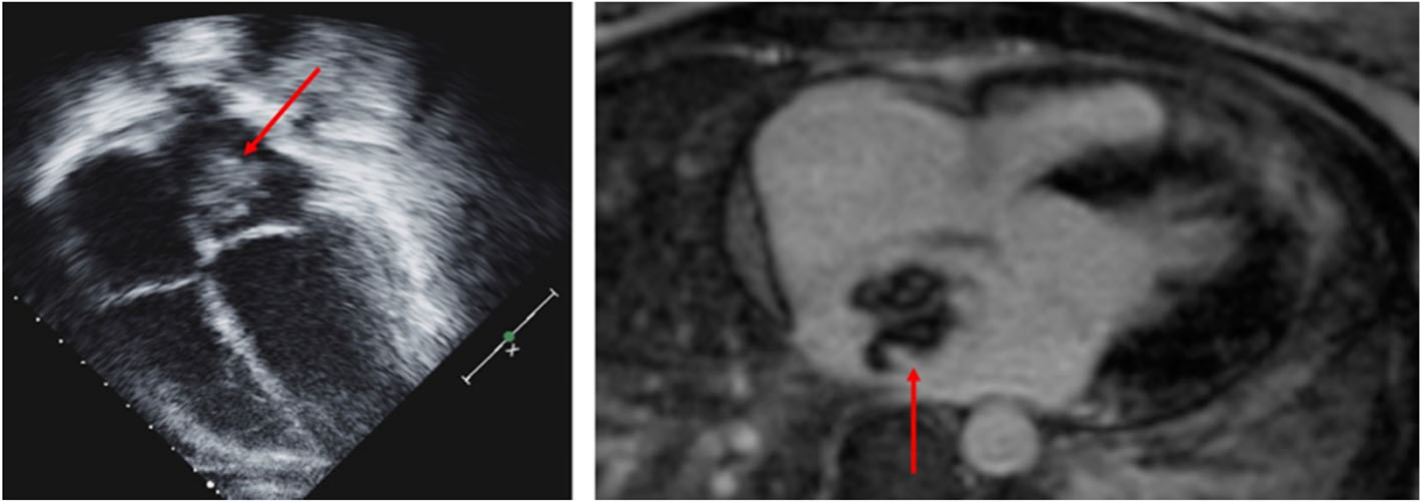
Echocardiographic image in the apical four chamber view shows a pedunculated mass attached to the atrial septum, red arrow. Cardiac magnetic resonance imaging stud, the axial four-chamber view shows the left atrial mass. Tissue characterization with T1 and T2 weighted images, first pass gadolinium perfusion and delayed enhancement sequences was highly suggestive of a myxoma

An echocardiogram was performed to screen for intra-cardiac thrombus and assess for coronary abnormalities due to concerns for vasculitis. The echocardiogram revealed a 2.5 by 3-cm (cm) mass in the left atrium (LA), Fig. 2. Cardiac magnetic resonance was performed for tissue characterization of the LA mass [7] and showed a 2.7 × 2.3 cm lobulated and mobile mass within the left atrium adherent to the septum (Fig. 2), consistent with a left atrial myxoma.

The patient’s rash and constitutional symptoms were attributed to the myxoma. Purpura and pain in the right toe was felt to be secondary to either micro-embolic phenomenon or vasospasm. The decision was made to proceed with surgical excision of the cardiac mass. She was observed off anticoagulation given the lack of any concerning neurologic symptoms until surgery, which followed shortly thereafter.

**Fig. 3:**
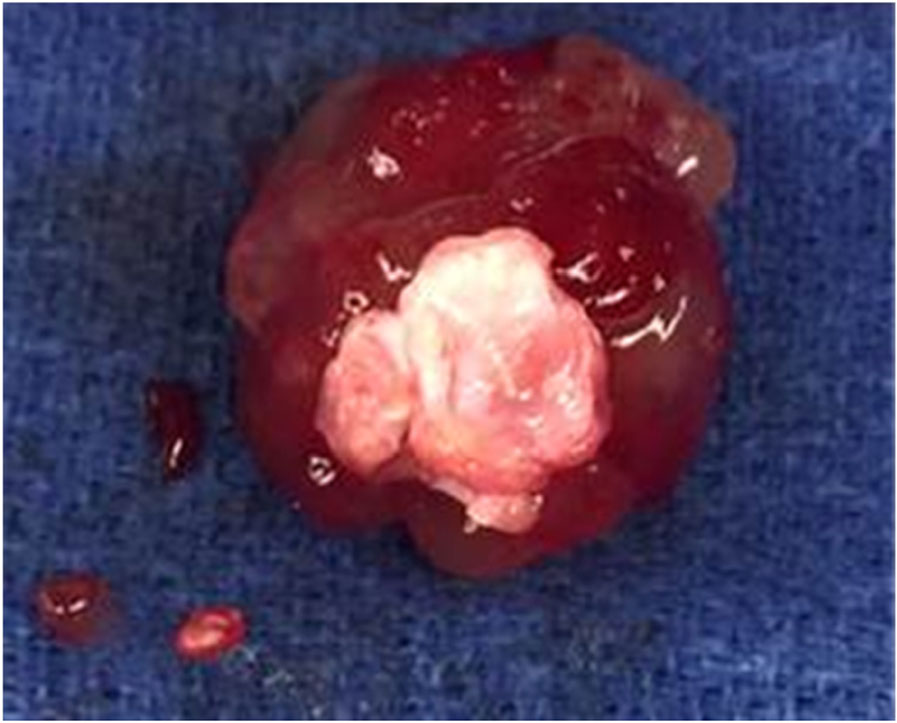
3.5 X 3.5 X 2.5 lobular, gelatinous, myxomatous mass following resection from left atrium in the operating room. Microscopic examination substantiated the diagnosis of a myxoma

**Fig. 4:**
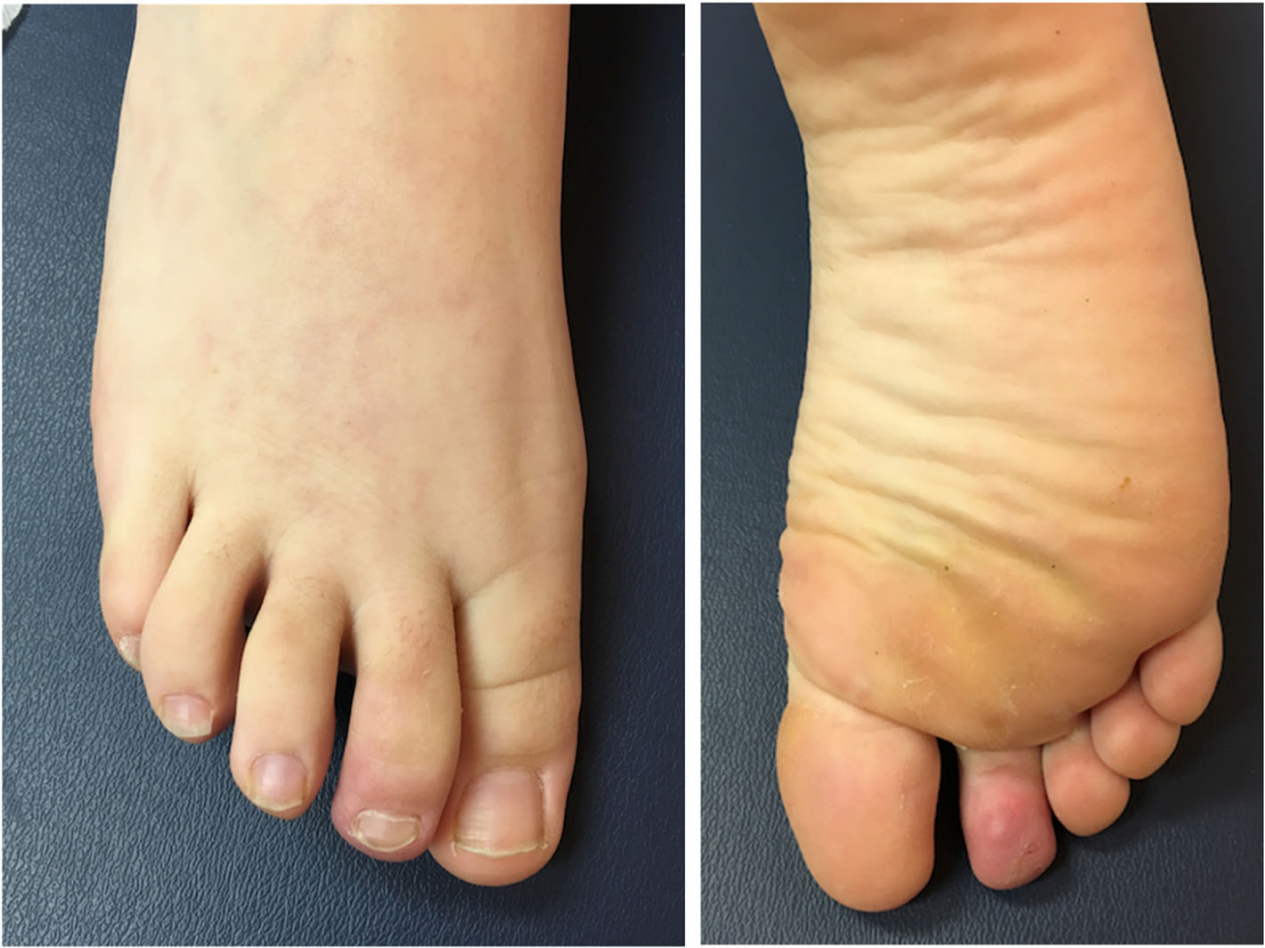
Resolution of purpura with minimal residual erythema at the right second toe 3 weeks after resection

The patient underwent an uncomplicated surgical resection and the diagnosis of cardiac myxoma was confirmed histologically (Fig. 3). She had an uncomplicated post-operative course with resolution of fevers and systemic symptoms in the immediate post-operative period. At 3-week follow-up she continued to be afebrile with near complete resolution of her skin findings (Fig. 4). She had no concerning symptoms at her 8-month follow up.

## Discussion and conclusions

Cardiac tumors are rare in children with an incidence of 0.17–0.2% [8, 9]. The most common pediatric cardiac tumors are rhabdomyomas [1, 4, 5, 10–12], 10% of which are familial and associated with Carney complex [6]. Cardiac myxomas have been diagnosed more frequently with the advent of echocardiography [3] and are most commonly located in the left atrium [5]. In children, the mean age of diagnosis is 9–10 years [5, 13]. Delayed or undiagnosed cardiac myxomas can result in severe or fatal complications due to embolization of these friable tumors or cardiac obstruction [14, 15].

As illustrated by our patient’s recurrent fevers, fatigue, skin findings and elevated inflammatory markers, cardiac myxomas can present with variable clinical symptoms that mimic other conditions (Table 2). Extracardiac manifestations are often caused by embolic phenomena and inflammation due to the intrinsic secretion of cytokines. In a series of 112 cases in adults, 34% of patients presented with systemic or constitutional symptoms and 16% presented with embolic manifestations [3]. However, review of the literature reveals only 6 reported cases of pediatric cardiac myxomas presenting with systemic symptoms without cardiac manifestations [15–21] and no cases of distal emboli without concurrent neurologic symptoms from cerebral emboli. Our case’s unique presentation with both systemic symptoms and vasculopathy (possibly due to emboli or vasospasm) highlights the importance of early recognition of the many features of these tumors to prevent to morbidity and mortality related to emboli.

A case series of children with cardiac myxomas causing cerebral emboli emphasizes the need for expedient diagnosis of cardiac myxomas; 6 out of 9 of these children had residual neurologic deficits and 1 died post-operatively [22]. Several of these children had distal extremity skin lesions noted before their neurologic events, demonstrating that earlier diagnosis is possible with an elevated index of suspicion. Interestingly, none of these cases had associated systemic symptoms or significantly elevated inflammatory markers as our patient did, indicating that we cannot attribute all of her symptoms to embolic phenomena and consider inflammation intrinsic to the myxoma as well (Table 3).

**Table 3:** Review of non-cardiac presentations of cardiac myxomas in the pediatric literature

Reference	Number of patients; age (years)/Gender	Systemic symptoms	Embolic signs	Elevated IL-6
Xu [25]	1;13/F	None	Headache	NA
Goldberg [15]	1; 3/M	None	Right hemiparesis, red spots on foot; died	NA
Omeroglu [26]	2; 4/F, 6/F	None	Stroke	NA
Al-Mateen [22]	2; 11/F, 10/M	None	Acute hemiplegia, transient ischemic attack, red spots	NA
Hovels [16]	1; 6/F	Fever, arthralgia	None	yes
Park [17]	1; 5/M	Vasculitis	None	NA
Shiraishi [18]	1	Fever	none	NA
Patel [20]	1;17 /M	Fatigue; rash	none	NA
Kaminsky [21]	1;14/M	Arthropathy	None	NA
Saji [19]	1	NA	NA	NA
Domanski [27]	1; 8/M	None	Stroke	NA
Tipton [28]	1; 17/M	None	Right hemiparesis, lethargy	NA
Bobo [29]	1; 15/F	None	Right hemiparesis, headache	NA
Tonz [30]	1; 8/M	None	Right hemiparesis, seizures, aphasia, red spots, retinal artery occlusion	NA
Hung [31]	1; 10/F	None	Right hemiparesis, retinal artery occlusion	NA
Bayir [32]	1; 14/F	None	Right hemiparesis, aphasia, slurred speech, cool right leg	NA
Landers [33]	1; 8/F	None	Right hemiparesis, expressive aphasia, pulmonary embolus	NA
Macias	1:13/F	Fever, fatigue	Purpura/distal emboli	No

Embolic signs include stoke, purpura, retinal artery occlusion. Systemic symptoms include fever, arthralgia, and fatigue. *NA* not applicable as not evaluated or not mentioned in work up

Cardiac myxoma is known to secrete pro-inflammatory cytokine interleukin 6 (IL-6) and serum IL-6 plasma level is correlated with constitutional symptoms [23]. Serum IL-6 level was normal in our case but this was examined after IV methylprednisolone was initiated. A previous report demonstrated that 11 out of 12 patients (92%) with cardiac myxoma related vasculitis experienced improvement of symptoms with steroid administration, thus her normal IL-6 level likely reflected prior steroid therapy [2] .

Paraneoplastic vasculitis is an under recognized diagnosis, occurring in 5.2% of vasculitis cases [24]. Given the rarity of the concurrent conditions, diagnosis of malignancy is often delayed and the mean interval before diagnosis of malignancy is reported to be 11.9 months. A diagnosis of vasculitis requires rheumatological and infectious investigation, but search for malignancy is mandatory when clinical course becomes chronic and refractory to conventional therapy. While there have been several case series of cardiac myxomas mimicking vasculitis in adults and 2 reports in children nd 2 reports in children [2], our patient did not have any evidence of vasculitis on biopsy, making it unlikely that systemic inflammation was the etiology of our patient’s distal skin findings.

**Fig. 5:**
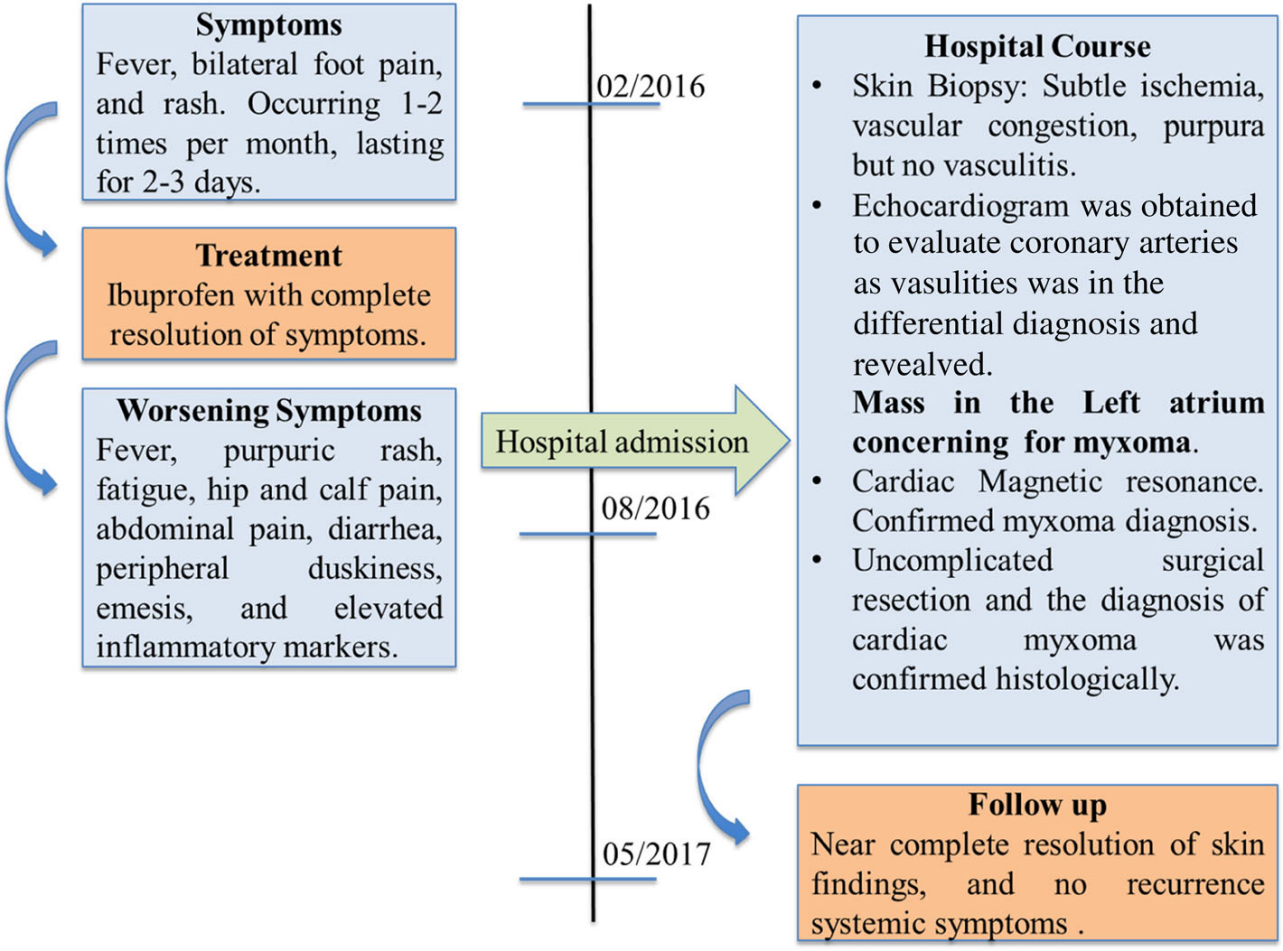
Timeline

In our case, systemic vasculitis and vasculopathy were the initial diagnostic considerations given our patient’s systemic symptoms and distal purpura. An echocardiogram quickly raised high suspicion for cardiac myxoma, supported by tissue characterization by MRI. This allowed for expedient tumor resection and improvement of symptoms (Fig. 5). This case emphasizes the importance of considering a cardiac myxoma in a patient with both systemic inflammation and vasculopathic phenomena.

## Abbreviations

ALT: Alanine transaminase
AST: Aspartate transaminase
CRP: C-reactive protein
ESR: Erythrocyte sedimentation rate
GGT: Gamma-glutamyl transferase
IL: Interleukin
LDH: Lactate dehydrogenase
